# Comparative efficacy and safety of immunotherapy for patients with advanced or metastatic esophageal squamous cell carcinoma: a systematic review and network Meta-analysis

**DOI:** 10.1186/s12885-022-10086-5

**Published:** 2022-09-17

**Authors:** Tian-Tian Gao, Jia-Hui Shan, Yu-Xian Yang, Ze-Wei Zhang, Shi-Liang Liu, Mian Xi, Meng-Zhong Liu, Lei Zhao

**Affiliations:** 1 Department of Radiation Oncology, Sun Yat-sen University Cancer Center, State Key Laboratory of Oncology in South China, Collaborative Innovation Center of Cancer Medicine, Guangzhou, China; 2 Department of Medicine, Qingdao University, Qingdao, Shandong China; 3 Department of Radiation Oncology, Sun Yat-sen University Cancer Center, State Key Laboratory of Oncology in South China, Guangdong Esophageal Cancer Institute, Collaborative Innovation Center for Cancer Medicine, Guangzhou, China

**Keywords:** Esophageal squamous cell carcinoma, Advanced or metastatic, Immunotherapy, Network meta-analysis

## Abstract

**Background:**

The study aimed to compare efficacy and safety of various immune checkpoint inhibitors for patients with advanced or metastatic esophageal squamous cell carcinoma (ESCC).

**Methods:**

We searched Medline, Web of Science, Cochrane Central Register of Controlled Trials, Embase, Clinical Trials.gov and several international conference databases from January 1, 2000 to December 19, 2021. We conducted Bayesian network meta-analysis to assess the relative effects among treatments. Outcomes included overall survival (OS), progression-free survival (PFS), overall response rate and adverse events.

**Results:**

Ten eligible trials with 5250 patients were included. Toripalimab and Camrelizumab plus chemotherapy were preferred to rank first on OS (probability, 61%) and PFS (probability, 37%) in the first-line setting, respectively. In refractory patients, Sintilimab and Camrlizumab were most likely to be ranked first on OS (probability, 37%) and PFS (probability, 94%). The toxicity related to immunotherapy was manageable in clinical trials. Camrelizumab and Nivolumab had the less adverse events of grade 3 or higher in the first and refractory setting, respectively.

**Conclusions:**

This study found that Toripalimab and Camrelizumab plus chemotherapy were likely to be the best option in terms of OS and PFS in the first-line setting for patients with advanced or metastatic ESCC respectively. Sintilimab and Camrelizumab were the preferred options for OS and PFS in refractory patients respectively. The toxicity of immunotherapy was different from conventional chemotherapy, but manageable in patients with ESCC.

**Trial registration:**

PROSPERO registration number: (CRD 42021261554).

**Supplementary Information:**

The online version contains supplementary material available at 10.1186/s12885-022-10086-5.

## Background

Esophageal cancer is the seventh most common cancer and ranks sixth for cancer-related mortality worldwide, with squamous-cell carcinoma (ESCC) accounting for approximately 85% of cases [1, 2]. Many esophageal cancers are advanced or metastatic at diagnosis. Combination fluoropyrimidine plus platinum-based chemotherapy and single-agent chemotherapy are recommended as the first-line and second-line treatments for patients with advanced or metastatic esophageal cancer, respectively. However, the overall survival of this population remains poor with an estimated median of 12 months [3–6]. Immune checkpoint inhibitors have shown significant survival benefits for patients with advanced esophageal cancer in several clinical trials [7–16].

Immune checkpoint inhibitors, such as programmed death receptor 1 (PD-1) or its ligand (PD-L1) inhibitor and anti-cytotoxic T-lymphocyte antigen 4 (CTLA-4) antibody, have been proposed in multiple solid carcinomas [17–19]. Blocking the immune checkpoint pathway can promote T-cell migration, proliferation, secretion of cytotoxic mediators and enhance immune response to cancer [20]. Various immune checkpoint inhibitors have shown effective antitumor activity in advanced or metastatic esophageal squamous cell carcinoma. However, there is no empirical evidence for the comparison of efficacy and safety among the different immune checkpoint inhibitors.

In the present study, we performed this network meta-analysis of randomized controlled trials to investigate the relative efficacy and safety of immune checkpoint inhibitors in the first-line and second-line treatments for patient with advanced or metastatic esophageal squamous cell carcinoma, respectively.

## Materials and methods

This network meta-analysis was performed following the PRISMA (preferred reporting items for systematic reviews and meta-analyses) extension statement (Supplementary Table 1) [21]. We used Bayesian network meta-analysis as its superiority to account for the effect of study-specific covariates and to generate precise evaluation using the present limited information. The protocol was registered with the Prospective Register of Systematic Reviews (PROSPERO CRD 42021261554).

### Data sources and search

Medline, Web of Science, Cochrane Central Register of Controlled Trials, Embase, and Clinical Trials.gov databases were searched for studies from January 1, 2000 to December 19, 2021 using a combination of main search terms: “esophageal carcinoma”, “advanced” and “metastatic” within the restriction limit of randomized controlled trials (RCTs). References from articles, commentaries, editorials, included studies and conference publications of relevant medical societies were hand-searched to ensure the completeness.

### Study selection

**Fig. 1:**
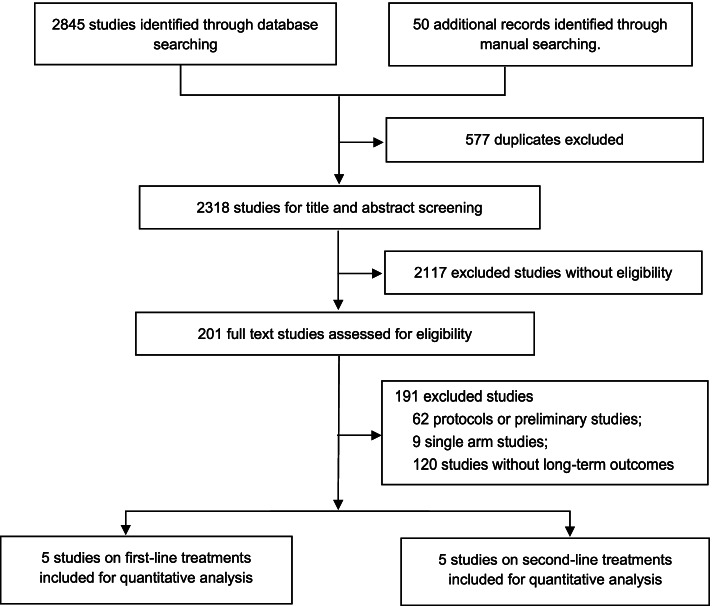
Study selection

We included randomized controlled trials which met the following criteria: 1) patients with histologically and clinically confirmed advanced or metastatic ESCC; 2) receiving at least one immune checkpoint inhibitor treatment; 3) reporting at least one of the following clinical outcomes: overall survival (OS), progression-free survival (PFS), overall response rate (ORR), adverse events of grade 3 or higher; 4) HER-2 expression negative status. We excluded protocols, preliminary studies, single-arm trials and studies not adhering to the inclusion criteria. The articles not published in English were also excluded. The titles and abstracts were sequentially screened, and the full text of potentially eligible articles was assessed for final inclusion (Fig. 1).

### Data extraction and risk of bias assessment

Data on trial details (e.g., study ID, size, patient age, number, patient gender), treatments (interventional and controlled arms) and primary outcomes were extracted into a spreadsheet. Two investigators conducted the study selection and data extraction independently to avoid potential assessment bias. Any discrepancies were resolved by another investigator. We preferred to use treatment-related adverse events, but all adverse events were accepted if they were not specified as treatment-related. For multiple publications on the same cohort, both the first report and the most recent publication were selected and the most recent data were used for the analysis. For conference abstracts and studies without complete outcomes, we contacted the trial investigators to request the final report; if unavailable, we extracted the data from ClinicalTrials.gov. Furthermore, some necessary survival data were obtained from the Kaplan-Meier curves [22–24].

We assessed each study’s risk of bias using the Cochrane Risk of Bias Tool, [25] which is based on the following domains: random sequence generation, allocation concealment, participants and personnel blinding, outcome assessment blinding, incomplete outcome data, selective outcome reporting, and other sources of bias. The studies were evaluated and scored as having low, high or unclear risk of bias (Supplementary Fig. 1). Two investigators assessed the risk of bias independently. Any discrepancy was resolved by a panel of adjudicators. We assessed the overall certainty of evidence for each outcome using the Grading Recommendations Assessment, Development and Evaluation (GRADE) approach and used the Guideline Development Tool (https://www.gradepro.org) to formulate the Summary of Findings table (Supplementary Table 2).

### Data synthesis and statistical analysis

To compare the efficacy and safety of different immune checkpoint inhibitors as first-line and second-line treatments in patients with advanced or metastatic ESCC, we synthesized all relative evidence in a Bayesian framework, such as hazard ratios (HR) for survival outcomes (OS and PFS) with the corresponding 95% credible intervals (95% CI) and odds ratios (OR) for binary outcomes (grade ≥ 3 adverse events and ORR). The primary outcomes were overall survival and progression-free survival. Secondary outcomes were overall response rate and adverse events of grade 3 or higher. We demonstrated the efficacy and safety of the different PD-1/L1inhibitors with network diagrams of comparisons, pooled estimates of the NMA, radar map, Bayesian ranking profiles and frequency toxicity profiles.

We conducted the separate network meta-analyses for OS, PFS, ORR and grade ≥ 3 adverse events using a fixed-effect consistency model in a Bayesian framework. We used noninformative uniform and normal prior distributions with four different sets of initial values to fit the model. For each outcome, 80,000 sample iterations were generated with 20,000 burn-ins and a thinning interval of 10. We evaluated convergence of iterations in accordance with the Brooks-Gelman-Rubin diagnostic (Supplement Fig. 2). When assessing OS and PFS, we applied contrast-based analyses using estimated differences in log HR and standard error calculated from published HR and their CIs [26]. The approach suggested in Woods et al. was adopted [27]. For the assessment of ORR and adverse events of grade 3 or higher, we applied arm-based analyses to estimate ORs and their CIs using the raw data presented in the manuscript. For each outcome, we estimated the relative ranking of the different immune checkpoint inhibitors according to the distribution of the ranking probabilities and the surface under the cumulative ranking (SUCRA).

Primary analysis for each outcome were performed using the most recent follow up data. Subgroup analysis was conducted based on the expression of PD-L1 according to the combined positive score (CPS) or tumor proportion score (TPS). Separate meta-analysis for OS and PFS were also conducted using a fixed-effect consistency model with a Bayesian approach for each group.

All analyses described were conducted using the ‘GeMtc’ package in R 4.1.3 (www.r-project.org).

## Results

### Study selection

The literature search identified 2895 publications. A total of 2318 articles were eligible for assessment after removing the duplicates and 2117 publications were excluded after screening the titles and abstracts. The full-text review was performed for 201 articles. Based on the selection criteria, 10 clinical trials (five studies for the first-line treatment and five studies for the second-line treatment) with a total of 5250 patients were deemed eligible for analysis (Fig. 1).

### First-line treatments

#### Characteristics of included trials

**Fig. 2:**
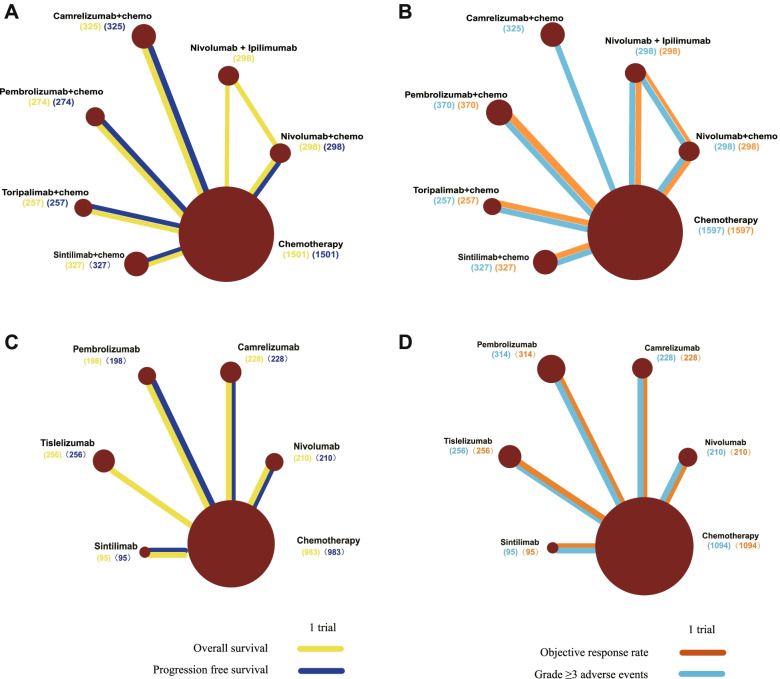
Network diagrams of comparison of different outcomes for patients with advanced or metastatic ESCC. **A** Comparison on OS and PFS in the first-line treatments. **B** Comparison of overall response rate and adverse events of 3 or higher in the first-line treatments. **C** Comparison on OS and PFS in the second-line treatments. **D** Comparison on overall response rate and adverse events of 3 or higher in the second-line treatments. Each circular node represents a type of treatment. The node size is proportional to the total sample size of the corresponding treatment in all studies. Each line represents a type of head-to-head comparison. The line width is proportional to the number of trials comparing the connected treatments. Abbreviations: ESCC, esophageal squamous cell carcinoma; chemo, chemotherapy

The five trials encompassed a total of 3280 patients with advanced or metastatic ESCC to receive 7 different treatments in the first-line setting (Nivolumab plus chemotherapy, Nivolumab plus Ipilimumab, Camrelizumab plus chemotherapy, Pembrolizumab plus chemotherapy, Toripalimab plus chemotherapy, Sintilimab plus chemotherapy and chemotherapy). KEYNOTE-590 enrolled patients with advanced or metastatic esophageal cancer or gastro-esophageal junction adenocarcinoma and we extracted the published data of patients with ESCC. The networks are presented in Fig. 2. The main characteristics of included studies are outlined in Table 1.

**Table 1:** Baseline characteristics of studies included in the network meta-analysis

Study	Size	Age	Male (%)	Reported outcomes	Interventional arm	Control arm
***First-line (n = 5)***						
Checkmate648	321/325/324	64/63/64	79/83/85	OS; PFS; ORR; Grade ≥ 3 AEs	Nivolumab 240 mg every 2 weeks plus chemotherapy (PF)	chemotherapy alone (Fluorouracil: 800 mg mg/m^2^ on days 1–5 plus cisplatin: 80 mg/m^2^ on days 1 for every 4 weeks)
				OS; ORR; Grade ≥ 3 AEs	Nivolumab (3 mg per kilogram every 2 weeks) plus Ipilimumab (1 mg per kilogram every 6 weeks)	
ESCORT-1st	298/298	62/62	87/88	OS; PFS; Grade ≥ 3 AEs	Camrelizumab 200 mg every 3 weeks plus chemotherapy (TP)	Placebo plus chemotherapy (Paclitaxel: 175 mg/m^2^ plus cisplatin: 75 mg/m^2^ on day 1 for every 3 weeks)
KEYNOTE 590	274/274	64/62	82/85	OS; PFS; ORR; Grade ≥ 3 AEs	Pembrolizumab 200 mg every 3 weeks plus chemotherapy (PF)	Placebo plus chemotherapy (Fluorouracil: 800 mg/m^2^ on days 1–5 plus cisplatin: 80 mg/m^2^ on day 1 for every 3 weeks)
JUPITER-06	257/257	63/62	84/86	OS; PFS; ORR; Grade ≥ 3 AEs	Toripalimab 240 mg every 3 weeks plus chemotherapy (TP)	Placebo plus chemotherapy (Paclitaxel: 175 mg/m^2^ plus cisplatin: 75 mg/m^2^ on day 1 for every 3 weeks)
ORIENT-15	327/322	/	/	OS; PFS; ORR; Grade ≥ 3 AEs	Sintilimab 200 mg every 3 weeks plus chemotherapy (TP or PF)	Placebo plus chemotherapy (Paclitaxel: 87.5 mg/m^2^ on day 1, 8 or 175 mg/m2 on day 1 plus cisplatin:75 mg/m^2^ on day 1; or fluorouracil: 800 mg/m2 on days 1–5 plus cisplatin:75 mg/m2 on day 1)
***Second-line (n = 5)***						
ATTRACTION-3	210/209	64/67	85/89	OS; PFS; ORR; Grade ≥ 3 AEs	Nivolumab 240 mg given every 2 weeks.	Investigator’s choice of chemotherapy (Paclitaxel 100 mg/m^2^ per week or docetaxel 75 mg/m^2^ every 3 weeks)
ESCORT	228/220	60/60	91/87	OS; PFS; ORR; Grade ≥ 3 AEs	Camrelizumab 200 mg given every 2 weeks.	Investigator’s choice of chemotherapy (Docetaxel 75 mg/m^2^ every 3 weeks or irinotecan180 mg/m^2^ every 2 weeks)
KEYNOTE-181	314/314	63/62	86.9/86.3	OS; PFS; ORR; Grade ≥ 3 AEs	Pembrolizumab 200 mg given every 3 weeks.	Investigator’s choice of chemotherapy (Paclitaxel 80–100 mg/m^2^ every 4 weeks, docetaxel 75 mg/m^2^ every 3 weeks or irinotecan 180 mg/m^2^ every 2 weeks)
RATIONALE 302	256/256	62/63	84.8/84.0	OS; ORR; Grade ≥ 3 AEs	Tislelzumab 200 mg given every 3 weeks.	Investigator’s choice of chemotherapy (Paclitaxel 135–175 mg /m^2^ every 3 weeks, docetaxel 75 mg/m^2^ every 3 weeks or irinotecan 125 mg/m^2^ every 3 weeks
ORIENT-2	95/95	59/59	92.6/88.4	OS; PFS; ORR; Grade ≥ 3 AEs	Sintilimab 200 mg given every 3 weeks.	Investigator’s choice of chemotherapy (Paclitaxel 175 mg/m^2^ every 3 weeks or irinotecan 180 mg/ m^2^ every 2 weeks)

*Abbreviations*: *OS* Overall survival, *PFS* Progression-free survival, *ORR* Overall response rate, *AEs* Adverse events, *PF* Fluorouracil and cisplatin, *TP* Paclitaxel and cisplatin

#### Network Meta-analysis

**Fig. 3:**
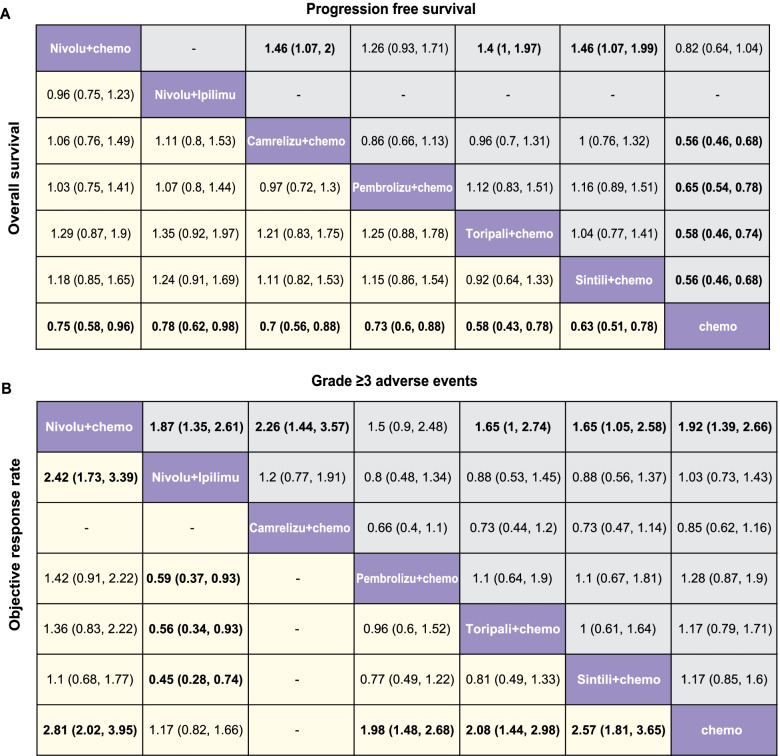
Pooled estimates of the network meta-analysis in the first-line setting. **A** Pooled HRs (95% CIs) for PFS (upper triangle) and OS (lower triangle). **B** Pooled OR (95% CIs) for adverse events of grade 3 or higher (upper triangle) and overall response rate (lower triangle). Data in each cell are HRs or ORs (95% CIs) for comparing row-defined versus column-defined treatment in the upper triangle, and for comparing column-defined treatment in the lower triangle. Significant results are in bold. Abbreviations: HR, hazard ratio; CI, confidence interval; OR, odd ratio; Nivolu, Nivolumab; Ipilimu, Ipilimumab; Camrelizu, Camrelizumab; Pembrolizu, Pembrolizumab; Toripali, Toripalimab; Sintili, Sintilimab; chemo, chemotherapy

**Fig. 4:**
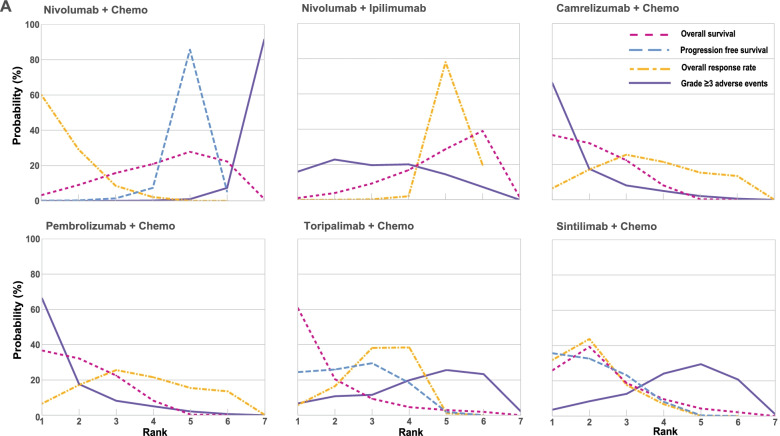
Bayesian ranking profiles of comparable treatments for efficacy and safety in the first-line setting. Profiles indicate the probability of each comparable treatment being ranked from first to last for OS, PFS, overall response rate and grade ≥ 3 adverse events. Ranking curves are described according to the Bayesian ranking results in Supplementary Table 2. Abbreviation: chemo, chemotherapy

In the network meta-analysis, we found that immune checkpoint inhibitors showed significant OS and PFS benefits over chemotherapy except Nivolumab plus chemotherapy on PFS (hazard ratio, 0.82; 95% credible interval, 0.64–1.04) (Fig. 3, Supplementary Fig. 3). Toripalimab and Camrelizumab in combination with chemotherapy were most likely to be ranked first for OS (probability, 61%) and PFS (probability, 37%), respectively (Fig. 4, Supplementary Table 2).

In terms of ORR, Nivolumab plus chemotherapy had the highest probability to obtain objective response for patients with advanced or metastatic ESCC (probability, 60%) (Fig. 4, Supplementary Table 3). The immune checkpoint inhibitors had manageable safety profile compared with chemotherapy, but the characteristics of toxicity were divergent. For example, adverse events related to immunotherapy consist of hypothyroidism, rash, hyponatremia and pneumonia which may range in severity from very mild to death. Of note, reactive capillary endothelial proliferation is a specific adverse event of Camrelizumab with approximately 80% incidence. The toxicities of chemotherapy are mainly present as anemia, neutropenia, leukopenia, nausea and decreased appetite (Supplementary Fig. 4). The rank of treatments according to SUCRA was summarized in Supplementary Table 4.

#### Subgroup analysis

We compared the efficacy of immune checkpoint inhibitors in the first-line setting according to the PD-L1 status: high expression group (CPS ≥10 or TPS ≥1) and low expression group (CPS < 10 or TPS < 1). We found that, immunotherapy could achieve OS benefits regardless of PD-L1 expression in advanced ESCC but not PFS benefits, that means a lack of PFS benefits did not rule out long-term OS benefits (Supplementary Fig. 5). Data for PD-L1 high expression group were available for all the treatments and Nivolumab plus chemotherapy showed the highest probability to be the best treatment in terms of OS while Camrelizumab plus chemotherapy ranked the highest in terms of PFS. For PD-L1 low expression group, Toripalimab plus chemotherapy ranked the highest for both OS and PFS (Supplementary Fig. 6).

### Second-line treatments

#### Characteristics of included trials

The five trials with a total of 1970 patients were identified in the second-line setting. All trials compared immune checkpoint inhibitors (Nivolumab, Camrelizumab, Pembrolizumab, Tislelizumab, Sintilimab) with single-agent chemotherapy. We extracted the data of patients with ESCC in KEYNOTE-590 trial. The networks are presented in Fig. 2. The main characteristics of included studies are outlined in Table 1.

#### Network Meta-analysis

**Fig. 5:**
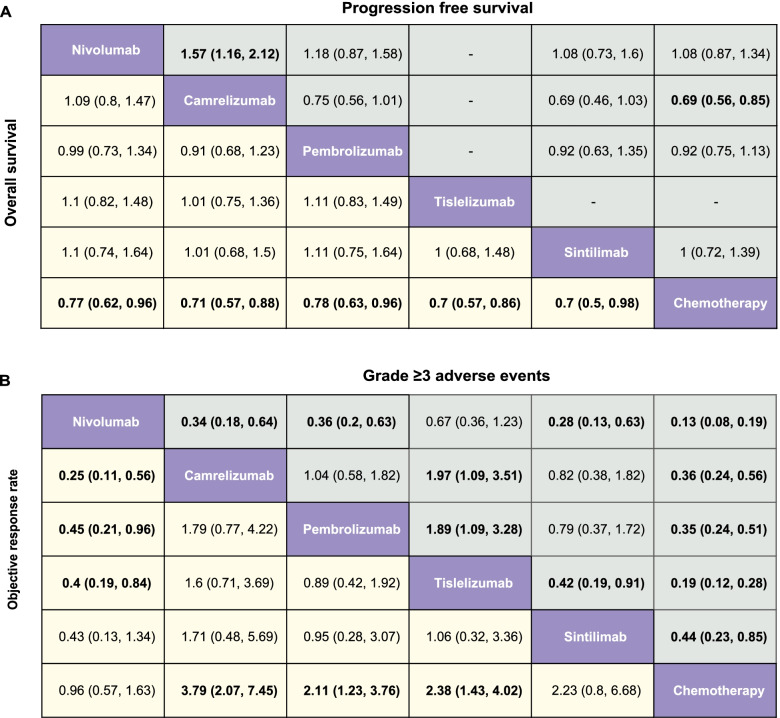
Pooled estimates of the network meta-analysis in the second-line setting. **A** Pooled HRs (95% CIs) for PFS (upper triangle) and OS (lower triangle). **B** Pooled OR (95% CIs) for adverse events of grade 3 or higher (upper triangle) and overall response rate (lower triangle). Data in each cell are HRs or ORs (95% CIs) for comparing row-defined versus column-defined treatment in the upper triangle, and for comparing column-defined treatment in the lower triangle. Significant results are in bold. Abbreviations: HR, hazard ratio; CI, confidence interval; OR, odd ratio; Nivolu, Nivolumab; Ipilimu, Ipilimumab; Camrelizu, Camrelizumab; Pembrolizu, Pembrolizumab; Toripali, Toripalimab; Sintili, Sintilimab; chemo, chemotherapy

**Fig. 6:**
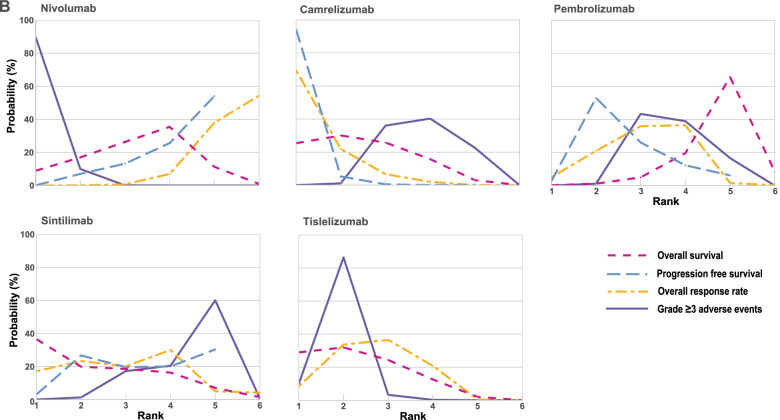
Bayesian ranking profiles of comparable treatments for efficacy and safety in the second-line setting. Profiles indicate the probability of each comparable treatment being ranked from first to last for OS, PFS, overall response rate and grade ≥ 3 adverse events. Ranking curves are described according to the Bayesian ranking results in Supplementary Table 2. Abbreviation: chemo, chemotherapy

In the network meta-analysis, all immune checkpoint inhibitors showed OS benefits compared with chemotherapy (Fig. 5, Supplementary Fig. 2). Sintilimab was likely to be ranked first (probability, 37%) in terms of OS (Fig. 6, Supplementary Table 2). Camrlizumab obtained statistically significant PFS benefit (HR, 0.69; 95% CI, 0.56–0.85) (Fig. 5) over chemotherapy and it was most likely to be ranked first in terms of PFS (probability, 94%) and ORR (probability, 69%) (Fig. 6, Supplementary Table 2). We found Nivolumab and Tislelizumab were consistent in causing less adverse events of grade 3 or higher (HR, 0.67; 95% CI, 0.36–1.23) (Fig. 5, Supplementary Fig. 2).

## Discussion

We performed a systematic review and network meta-analysis of the first-line and second-line immunotherapy in patients with advanced or metastatic ESCC. There are several important findings. First, Toripalimab and Camrelizumab in combination with chemotherapy were associated to the highest probability to be the best treatments in terms of OS and PFS in the first-line setting, respectively. Sintilimab and Camrelizumab were likely to be the preferred treatments for OS and PFS respectively in the second-line treatments (Supplementary Fig. 7). Second, we saw less toxicity related to immune checkpoint inhibitors than conventional chemotherapy. More than 50 different types of specific adverse events were reported, of which 16 were selected as a representation of the most clinically relevant in current practice. Commonly reported severe adverse events for immune checkpoint inhibitors included hypothyroidism, rash and pneumonia which were different from those of convention chemotherapy (decreased neutrophil count, anemia, neutropenia and nausea).

For appropriate interpretation, these findings require context. First, KEYNOTE590 and Checkmate648, [7, 8] the global phase 3 randomized controlled trials, have proven the significant efficacy of immune checkpoint inhibitors (Pembrolizumab and Nivolumab) for patients with advanced ESCC in the first-line setting. Based on these data, the United States Food and Drug Administration (FDA) approve Pembrolizumab plus chemotherapy as the first-line treatment in advanced esophageal cancer for patients with a CPS of ≥10, while Nivolumab in combination with chemotherapy or Ipilimumab were approved for first-line ESCC indications in all patients. In contrast, Camrelizumab, Toripalimab and Sintilimab were investigated in the ESCORT-1st, JUPITER-06 and ORIENT-16 trials, respectively, using paclitaxel and cisplatin as the chemotherapy backbone mainly in Asian corhort [9–11]. No trials were registered for these immune checkpoint inhibitors outside of Asia. Second, the evaluation of PD-L1 positivity seems to be important for effective treatment decisions. In the present study, PD-L1 high expression was characterized by tumor proportion score (TPS) > 1% (in Checkmate 648) or combined positive score (CPS) > 10% according to the included trials. Post hoc analysis in these clinical studies suggested a potentially better overall survival benefit in patients with positive PD-L1 expression, but the test for interaction was not statistically significant. Therefore, novel biomarkers, such as tumor mutational burden (TMB), and clinical characteristics for predicting the response to immunotherapy are needed to be further explored. Third, immunotherapy has been recommended as the first-line treatment for patient with advanced or metastatic ESCC, but primary or secondary immune resistance remains a major concern in patient management. Previous studies showed that patients with fewer lines of immunotherapy might have less refractory and immunosuppressive tumor microenvironments [28]. Strategies to overcome such immune resistance are underway, including the modification of tumor vasculature by targeting endothelial growth factor receptor (EGFR) or vascular endothelial growth factor receptor (VEGFR), combination with the lymphocyte activation gene-3 (LAG3) inhibitor as well as enhancing the tumor-specific T cells with personalized approaches such as CAR T cell therapy [29–31]. Fourth, we recognized that the preferred treatment option may differ according to the various endpoints. In fact, the choice of treatment is based not only on the demonstration of the superiority of one treatment over another but also on the toxicity profile. The present study showed adverse events related to immunotherapy were mild or transient, including hypothyroidism, rash and pneumonia. Of note, Camrelizumab was the most likely to cause reactive capillary endothelial proliferation, which could regress spontaneously after discontinuation of Camrelizumab. In general, the head-to-head comparison of similar combination of immunotherapy is hardly performed in the future, and our meta-analysis offers the appropriate response to the clinical issue regarding the choice of several similar therapeutic options.

For the unique mechanism of action regarding to immune checkpoint inhibitors, different endpoints on clinical researches should be interpreted in an appropriate way to capture the survival benefits accurately. The Kaplan-Meier (KM) estimator and the Cox proportional hazards model are standard methods for survival analysis in oncology drug development. In immunotherapy trials, however, long tails and crossovers in KM survival curves may violate the proportional assumption, making the efficacy of immune checkpoint inhibitors underestimated. For example, in Checkmate 648 trial, there was a cross-over of the survival curves in Nivolumab plus Ipilimumab arm for patients with advanced ESCC, indicating an early weaker effect with dual immunotherapy than conventional chemotherapy. But the long tail of the curves reflected a survival plateau of patients with durable responses, which may represent the maximum benefits of this chemotherapy-free regimen. Furthermore, previous studies have reported that PFS was a suitable surrogate for OS in patients with esophageal carcinoma treated with cytotoxic agents to complete clinical trials in an expeditious way [32]. However, the surrogacy of PFS or ORR was not validated in the setting of immunotherapy for patients with advanced esophageal carcinoma, possibly due to crossover and pseudo-progression. For example, Camrelizumab achieved the best PFS benefits in both the first-line and second-line treatments for patients with advanced ESCC, but may not convert to the best long-term OS benefit. Hence, the lack of PFS or ORR benefit did not rule out longer-term OS benefit, indicating the importance of understanding the biologic mechanisms of action when interpreting various outcomes in immunotherapy trials .

The present study had several limitations. First, unavoidable confounding factors remain in this inherently observation network meta-analysis based on data from clinical trials. This approach could be acknowledged as a surrogate for head-to-head treatment comparison as it is scarcely likely to conduct comparative clinical trials in real world. Second, the analysis of published aggregate data compared with individual patient data has limits to perform subgroup analysis and effect modification according to clinical characters. For example, we used survival data of subgroup with different PD-L1 status from included studies to compare the efficacy among immune checkpoint inhibitors. It was a potential source of uncertain randomization and heterogeneity of patients’ characteristic. Third, individual treatment administration in real world may differ from clinical trials which could influence the efficacy and toxicity. But the present analysis aimed to provide guidance for clinicians and patients to make appropriate treatment decisions. Fourth, the included studies examined only patients with advanced or metastatic ESCC, while data for patients with adenocarcinoma or other histology remains unknown. Thus, these results may not necessarily be extrapolated to them. Finally, questions regarding the efficacy and safety of immune checkpoint inhibitors in maintenance use was not investigated and therefore, remains a subject for future studies.

## Conclusions

In this systematic review and network meta-analysis of immunotherapy for patients with advanced ESCC, Toripalimab and Camrelizumab plus chemotherapy were identified to provide the greatest OS and PFS benefit in the first-line setting, respectively. Sintilimab and Camrelizumab were likely to be the preferred treatments for OS and PFS respectively in the second-line treatments. These findings could provide guidance to clinicians and patients when making treatment decisions. Future trials may focus on other potential combinations and sequencing of immunotherapy in patients with advanced ESCC.

## Supplementary Information

**Additional file 1.**

**Additional file 2.**

**Additional file 3.**

**Additional file 4.**

**Additional file 5.**

**Additional file 6.**

**Additional file 7.**

**Additional file 8.**

**Additional file 9.**

**Additional file 10.**

**Additional file 11.**

## Data Availability

The datasets used and/or analyzed during the current study are available from the corresponding author on reasonable request.

## Abbreviations

ESCC: Esophageal squamous cell carcinoma
OS: Overall survival
PFS: Progression-free survival
HR: Hazard ratio
CI: Credible interval
EGFR: Endothelial growth factor receptor
VEGFR: vascular endothelial growth factor receptor
PD-1: Programmed cell death 1
PD-L1: Programmed cell death 1 ligand-1
PRISMA: Preferred reporting items for systematic reviews and meta-analyses
OR: Odds ratios
ORR: Objective response rate
RECIST: Response Evaluation Criteria in Solid Tumors
TPS: Tumor proportion score
CPS: Combined positive score
