# Supplementary material for: Comparative efficacy and safety of immunotherapy for patients with advanced or metastatic esophageal squamous cell carcinoma: a systematic review and network Meta-analysis
Source: BMC Cancer. 2022 Sep 17;22:992. doi: 10.1186/s12885-022-10086-5 (PMC9482734; doi:10.1186/s12885-022-10086-5)
Supplement: Supplementary file 2 — Additional file 2. [file 12885_2022_10086_MOESM2_ESM.docx]

| **Supplementary Table 2. Summary of findings** | | | | | | |
| --- | --- | --- | --- | --- | --- | --- |
| **Immunotherapy plus chemotherapy compared to chemotherapy for advanced or metastatic esophageal squamous cell carcinoma in the first-line treatments.** | | | | | | |
| **Patient or population:** advanced or metastatic esophageal squamous cell carcinoma  **Intervention:** Immunotherapy plus chemotherapy  **Comparison:** chemotherapy | | | | | | |
| Outcome № of participants (studies) | Relative effect (95% CI) | **Anticipated absolute effects (95% CI)** | | | Certainty | What happens |
|  |  | **Chemo** | **Immuno+chemo** | **Difference** |  |  |
| OS № of participants: 3812 (5 RCTs) | **HR 0.70** (0.64 to 0.77) | 64.7% | **51.8%** (48.7 to 55.2) | **12.9% fewer** (16.1 fewer to 9.6 fewer) | ⨁⨁⨁⨁ High |  |
| PFS № of participants: 3174 (5 RCTs) | **HR 0.62** (0.56 to 0.68) | 65.0% | **47.8%** (44.4 to 51) | **17.2% fewer** (20.5 fewer to 14 fewer) | ⨁⨁⨁⨁ High |  |
| ORR № of participants: 3150 (4 RCTs) | **OR 2.03** (1.75 to 2.36) | 38.1% | **55.5%** (51.8 to 59.2) | **17.4% more** (13.8 more to 21.1 more) | ⨁⨁⨁⨁ High |  |
| AE № of participants: 3812 (5 RCTs) | **OR 1.19** (1.04 to 1.36) | 56.8% | **61.0%** (57.7 to 64.1) | **4.2% more** (1 more to 7.3 more) | ⨁⨁⨁⨁ High |  |
|  | | | | | | |
| **Immunotherapy compared to chemotherapy for patients with advanced oesophageal squamous cell carcinoma refractory or intolerant to previous chemotherapy** | | | | | | |
| **Patient or population:** patients with advanced oesophageal squamous cell carcinoma refractory or intolerant to previous chemotherapy  **Intervention:** Immunotherapy  **Comparison:** chemotherapy | | | | | | |
| Outcome № of participants (studies) | Relative effect (95% CI) | **Anticipated absolute effects (95% CI)** | | | Certainty | What happens |
|  |  | **Chemo** | **Immuno** | **Difference** |  |  |
| OS № of participants: 2197 (5 RCTs) | **HR 0.73** (0.66 to 0.81) | 86.1% | **76.3%** (72.8 to 79.8) | **9.8% fewer** (13.3 fewer to 6.3 fewer) | ⨁⨁⨁⨁ High |  |
| PFS № of participants: 1685 (4 RCTs) | **HR 0.9** (0.8 to 1.0) | 81.5% | **78.1%** (74.1 to 81.5) | **3.4% fewer** (7.4 fewer to 0 fewer) | ⨁⨁⨁⨁ High |  |
| ORR № of participants: 2197 (5 RCTs) | **OR 1.99** (1.54 to 2.58) | 9.1% | **16.7%** (13.4 to 20.6) | **7.5% more** (4.3 more to 11.5 more) | ⨁⨁⨁⨁ High |  |
| AE № of participants: 2197 (5 RCTs) | **OR 0.26** (0.21 to 0.31) | 47.3% | **19.0%** (15.9 to 21.8) | **28.4% fewer** (31.5 fewer to 25.5 fewer) | ⨁⨁⨁⨁ High |  |
| ***The risk in the intervention group** (and its 95% confidence interval) is based on the assumed risk in the comparison group and the **relative effect** of the intervention (and its 95% CI). **CI:** confidence interval; **HR:** hazard Ratio; **OR:** odds ratio | | | | | | |
| **GRADE Working Group grades of evidence** **High certainty:** we are very confident that the true effect lies close to that of the estimate of the effect. **Moderate certainty:** we are moderately confident in the effect estimate: the true effect is likely to be close to the estimate of the effect, but there is a possibility that it is substantially different. **Low certainty:** our confidence in the effect estimate is limited: the true effect may be substantially different from the estimate of the effect. **Very low certainty:** we have very little confidence in the effect estimate: the true effect is likely to be substantially different from the estimate of effect. | | | | | | |

[Abbreviation](javascript:;): Immuno, immunotherapy; Chemo, chemotherapy; OS, overall survival; PFS, progression-free survival; ORR, overall response rate; AE, adverse event; HR, hazard ratio; OR, odds ratio; RCT, randomized controlled trial.
