# Supplementary material for: Comparative efficacy and safety of immunotherapy for patients with advanced or metastatic esophageal squamous cell carcinoma: a systematic review and network Meta-analysis
Source: BMC Cancer. 2022 Sep 17;22:992. doi: 10.1186/s12885-022-10086-5 (PMC9482734; doi:10.1186/s12885-022-10086-5)
Supplement: Supplementary file 3 — Additional file 3. [file 12885_2022_10086_MOESM3_ESM.docx]

**Supplementary Table 3.** Bayesian ranking results of network meta-analysis.

| ***First-line*** | | | | | | | | | | | | |
| --- | --- | --- | --- | --- | --- | --- | --- | --- | --- | --- | --- | --- |
| **Treatments** | **Rank of possibility(%)** | | | | | | | | | | | |
|  | **1** | **2** | | **3** | | **4** | | **5** | | **6** | | **7** |
| **Overall survival** | | | | | | | | | | | | |
| Nivolu + chemo | 3 | 9 | | 16 | | 21 | | 28 | | 22 | | 1 |
| Nivolu + Ipilimu | 1 | 4 | | 9 | | 17 | | 29 | | 39 | | 2 |
| Camrelizu + chemo | 7 | 17 | | 26 | | 22 | | 15 | | 14 | | 0 |
| Pembrolizu + chemo | 3 | 10 | | 21 | | 27 | | 21 | | 18 | | 0 |
| Toripali + chemo | **61** | 20 | | 9 | | 5 | | 3 | | 2 | | 0 |
| Sintili + chemo | 26 | 39 | | 19 | | 10 | | 4 | | 2 | | 0 |
| chemo | 0 | 0 | | 0 | | 0 | | 0 | | 3 | | **97** |
| **Progression-free survival** | | | | | | | | | | | | |
| Nivolu + chemo | 0 | 0 | | 1 | | 7 | | 86 | | 5 | | - |
| Camrelizu + chemo | **37** | 32 | | 22 | | 8 | | 0 | | 0 | | - |
| Pembrolizu + chemo | 3 | 9 | | 23 | | 58 | | 6 | | 0 | | - |
| Toripali + chemo | 24 | 26 | | 30 | | 18 | | 2 | | 0 | | - |
| Sintili + chemo | 36 | 33 | | 23 | | 8 | | 0 | | 0 | | - |
| chemo | 0 | 0 | | 0 | | 0 | | 5 | | **95** | | - |
| **Objective response rate** | | | | | | | | | | | | |
| Nivolu + chemo | **60** | 29 | | 9 | | 2 | | 0 | | 0 | | - |
| Nivolu + Ipilimu | 0 | 0 | | 0 | | 2 | | 78 | | 20 | | - |
| Pembrolizu + chemo | 2 | 11 | | 35 | | 51 | | 1 | | 0 | | - |
| Toripali + chemo | 6 | 16 | | 38 | | 38 | | 1 | | 0 | | - |
| Sintili + chemo | 32 | 44 | | 18 | | 7 | | 0 | | 0 | | - |
| chemo | 0 | 0 | | 0 | | 0 | | 20 | | **80** | | - |
| **Grade ≥3 adverse events** | | | | | | | | | | | | |
| Nivolu + chemo | 0 | 0 | | 0 | | 0 | | 1 | | 7 | | **92** |
| Nivolu + Ipilimu | 16 | 23 | | 20 | | 20 | | 14 | | 7 | | 0 |
| Camrelizu + chemo | **66** | 18 | | 8 | | 5 | | 2 | | 1 | | 0 |
| Pembrolizu + chemo | 2 | 6 | | 7 | | 15 | | 25 | | 41 | | 5 |
| Toripali + chemo | 7 | 11 | | 12 | | 20 | | 26 | | 23 | | 2 |
| Sintili + chemo | 4 | 8 | | 13 | | 24 | | 29 | | 21 | | 1 |
| chemo | 5 | 35 | | 41 | | 16 | | 3 | | 0 | | 0 |
| ***Second-line*** | | | | | | | | | | | | |
| **Treatments** | **Rank of possibility(%)** | | | | | | | | | | | |
|  | **1** | | **2** | | **3** | | **4** | | **5** | | **6** | |
| **Overall survival** | | | | | | | | | | | | |
| Nivolumab | 9 | | 17 | | 26 | | 35 | | 11 | | 1 | |
| Camrelizumab | 25 | | 30 | | 26 | | 16 | | 3 | | 0 | |
| Pembrolizumab | 0 | | 1 | | 5 | | 20 | | 66 | | 8 | |
| Tislelizumab | 29 | | 32 | | 24 | | 13 | | 2 | | 0 | |
| Sintilimab | **37** | | 20 | | 19 | | 16 | | 7 | | 2 | |
| chemotherapy | 0 | | 0 | | 0 | | 0 | | 11 | | **89** | |
| **Progression-free survival** | | | | | | | | | | | | |
| Nivolumab | 0 | | 7 | | 13 | | 26 | | **54** | | - | |
| Camrelizumab | **94** | | 5 | | 0 | | 0 | | 0 | | - | |
| Pembrolizumab | 3 | | 53 | | 26 | | 12 | | 6 | | - | |
| Sintilimab | 3 | | 27 | | 20 | | 20 | | 30 | | - | |
| chemotherapy | 0 | | 8 | | 41 | | 42 | | 9 | | - | |
| **Objective response rate** | | | | | | | | | | | | |
| Nivolumab | 0 | | 0 | | 1 | | 7 | | 38 | | **54** | |
| Camrelizumab | **69** | | 22 | | 7 | | 2 | | 0 | | 0 | |
| Pembrolizumab | 5 | | 21 | | 36 | | 36 | | 2 | | 0 | |
| Tislelizumab | 8 | | 34 | | 37 | | 21 | | 0 | | 0 | |
| Sintilimab | 17 | | 23 | | 20 | | 30 | | 5 | | 4 | |
| chemotherapy | 0 | | 0 | | 0 | | 4 | | 55 | | 41 | |
| **Grade ≥3 adverse events** | | | | | | | | | | | | |
| Nivolumab | **90** | | 10 | | 0 | | 0 | | 0 | | 0 | |
| Camrelizumab | 0 | | 1 | | 36 | | 40 | | 23 | | 0 | |
| Pembrolizumab | 0 | | 1 | | 43 | | 39 | | 17 | | 0 | |
| Tislelizumab | 10 | | 86 | | 3 | | 0 | | 0 | | 0 | |
| Sintilimab | 0 | | 1 | | 17 | | 21 | | 60 | | 1 | |
| chemotherapy | 0 | | 0 | | 0 | | 0 | | 1 | | **99** | |

Abbreviations: Nivolu, Nivolumab; Ipilimu, Ipilimumab; Camrelizu, Camrelizumab; Pembrolizu, Pembrolizumab; Toripali, Toripalimab; Sintili, Sintilimab; chemo, chemotherapy.
