# Supplementary material for: Comparative efficacy and safety of immunotherapy for patients with advanced or metastatic esophageal squamous cell carcinoma: a systematic review and network Meta-analysis
Source: BMC Cancer. 2022 Sep 17;22:992. doi: 10.1186/s12885-022-10086-5 (PMC9482734; doi:10.1186/s12885-022-10086-5)
Supplement: Supplementary file 4 — Additional file 4. [file 12885_2022_10086_MOESM4_ESM.docx]

**Supplementary Table 4.** The SUCRA ranking of network meta-analysis

|  |  | **Outcomes of Efficacy** | | | |  | **Outcomes of Activity** | |  | **Outcomes of Safety** | |
| --- | --- | --- | --- | --- | --- | --- | --- | --- | --- | --- | --- |
| **Treatments** |  | Rank OS | SUCRA | Rank PFS | SUCRA |  | Rank ORR | SUCRA |  | Rank AEs | SUCRA |
| ***First-line treatments*** |  |  |  |  |  |  |  |  |  |  |  |
| Toripalimab + chemotherapy |  | 1 | 87.40% | 3 | 70.35% |  | 3 | 57.57% |  | 4 | 45.59% |
| Sintilimab + chemotherapy |  | 2 | 77.66% | 2 | 79.05% |  | 2 | 80.30% |  | 5 | 44.40% |
| Camrelizumab + chemotherapy |  | 3 | 56.35% | 1 | 79.31% |  | / | / |  | 1 | 89.79% |
| Pembrolizumab + chemotherapy |  | 4 | 48.64% | 4 | 48.97% |  | 4 | 52.38% |  | 6 | 34.08% |
| Nivolumab + chemotherapy |  | 5 | 44.79% | 5 | 21.31% |  | 1 | 89.24% |  | 7 | 1.62% |
| Nivolumab + Ipilimumab |  | 6 | 34.67% | / | / |  | 5 | 16.60% |  | 3 | 64.09% |
| Chemotherapy |  | 7 | 0.50% | 6 | 1.02% |  | 6 | 3.91% |  | 2 | 70.43% |
| ***Second-line treatments*** |  |  |  |  |  |  |  |  |  |  |  |
| Nivolumab |  | 4 | 47.94% | 5 | 18.47% |  | 1 | 89.04% |  | 1 | 97.96% |
| Camrelizumab |  | 3 | 67.28% | 1 | 98.40% |  | 6 | 8.33% |  | 4 | 43.17% |
| Pembrolizumab |  | 5 | 45.60% | 2 | 58.40% |  | 5 | 41.84% |  | 3 | 45.67% |
| Tislelizumab |  | 1 | 70.51% | / | / |  | 4 | 33.92% |  | 2 | 81.25% |
| Sintilimab |  | 2 | 67.95% | 3 | 37.92% |  | 3 | 39.42% |  | 5 | 31.80% |
| Chemotherapy |  | 6 | 0.72% | 4 | 36.82% |  | 2 | 87.45% |  | 6 | 0.13% |
