# Supplementary material for: Comparative efficacy and safety of immunotherapy for patients with advanced or metastatic esophageal squamous cell carcinoma: a systematic review and network Meta-analysis
Source: BMC Cancer. 2022 Sep 17;22:992. doi: 10.1186/s12885-022-10086-5 (PMC9482734; doi:10.1186/s12885-022-10086-5)
Supplement: Supplementary file 5 — Additional file 5. [file 12885_2022_10086_MOESM5_ESM.pdf]

A

	Random sequence generation (selection bias)	Allocation concealment (selection bias)	Blinding of participants and personnel (performance bias)	Blinding of outcome assessment (detection bias)	Incomplete outcome data (attrition bias)	Selective reporting (reporting bias)	Other bias
Checkmate648	+	+	−	−	+	+	+
ESCORT-1st	+	+	+	?	+	+	+
JUPITER-06	+	+	+	?	+	+	+
KEYNOTE-590	+	+	+	?	+	+	+
ORIENT-15	+	+	+	?	+	+	+

B

	Random sequence generation (selection bias)	Allocation concealment (selection bias)	Blinding of participants and personnel (performance bias)	Blinding of outcome assessment (detection bias)	Incomplete outcome data (attrition bias)	Selective reporting (reporting bias)	Other bias
ATTRACTION-3	+	+	+	+	+	+	+
ESCORT	+	+	+	+	+	+	+
KEYNOTE-181	+	?	−	+	+	+	+
ORIENT-2	+	+	+	+	+	+	+
RATIONALE302	+	?	?	+	+	+	+
