# Supplementary material for: Comparative efficacy and safety of immunotherapy for patients with advanced or metastatic esophageal squamous cell carcinoma: a systematic review and network Meta-analysis
Source: BMC Cancer. 2022 Sep 17;22:992. doi: 10.1186/s12885-022-10086-5 (PMC9482734; doi:10.1186/s12885-022-10086-5)
Supplement: Supplementary file 7 — Additional file 7. [file 12885_2022_10086_MOESM7_ESM.pdf]

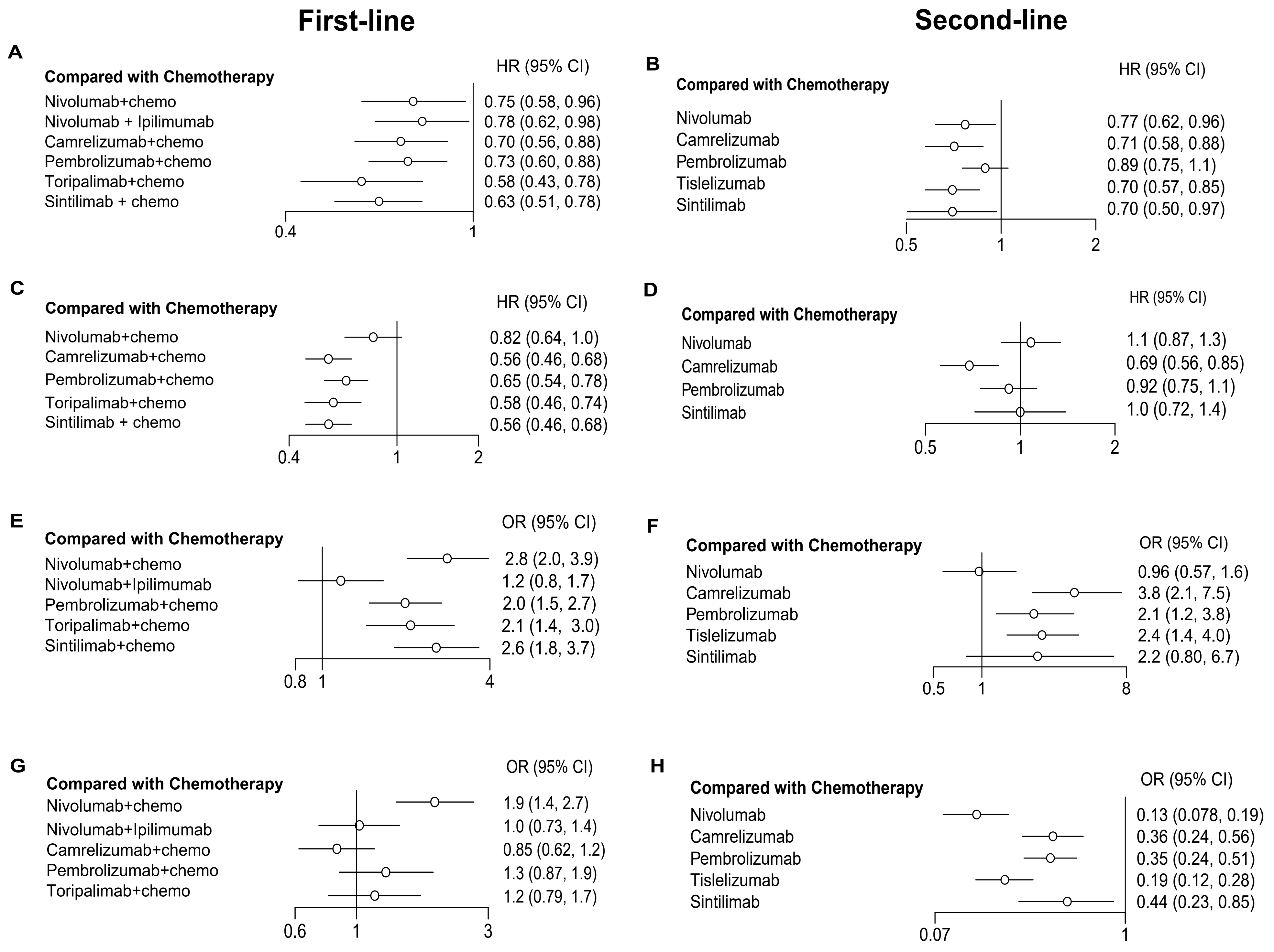
First-line
Second-line
A
Compared with Chemotherapy
HR (95% CI)
Nivolumab+chemo 0.75 (0.58, 0.96)
Nivolumab + Ipilimumab 0.78 (0.62, 0.98)
Camrelizumab+chemo 0.70 (0.56, 0.88)
Pembrolizumab+chemo 0.73 (0.60, 0.88)
Toripalimab+chemo 0.58 (0.43, 0.78)
Sintilimab + chemo 0.63 (0.51, 0.78)
0.4
1
B
Compared with Chemotherapy
HR (95% CI)
Nivolumab 0.77 (0.62, 0.96)
Camrelizumab 0.71 (0.58, 0.88)
Pembrolizumab 0.89 (0.75, 1.1)
Tislelizumab 0.70 (0.57, 0.85)
Sintilimab 0.70 (0.50, 0.97)
0.5
1
2
C
Compared with Chemotherapy
HR (95% CI)
Nivolumab+chemo 0.82 (0.64, 1.0)
Camrelizumab+chemo 0.56 (0.46, 0.68)
Pembrolizumab+chemo 0.65 (0.54, 0.78)
Toripalimab+chemo 0.58 (0.46, 0.74)
Sintilimab + chemo 0.56 (0.46, 0.68)
0.4
1
2
D
Compared with Chemotherapy
HR (95% CI)
Nivolumab 1.1 (0.87, 1.3)
Camrelizumab 0.69 (0.56, 0.85)
Pembrolizumab 0.92 (0.75, 1.1)
Sintilimab 1.0 (0.72, 1.4)
0.5
1
2
E
Compared with Chemotherapy
OR (95% CI)
Nivolumab+chemo 2.8 (2.0, 3.9)
Nivolumab+Ipilimumab 1.2 (0.8, 1.7)
Pembrolizumab+chemo 2.0 (1.5, 2.7)
Toripalimab+chemo 2.1 (1.4, 3.0)
Sintilimab+chemo 2.6 (1.8, 3.7)
0.8
1
4
F
Compared with Chemotherapy
OR (95% CI)
Nivolumab 0.96 (0.57, 1.6)
Camrelizumab 3.8 (2.1, 7.5)
Pembrolizumab 2.1 (1.2, 3.8)
Tislelizumab 2.4 (1.4, 4.0)
Sintilimab 2.2 (0.80, 6.7)
0.5
1
8
G
Compared with Chemotherapy
OR (95% CI)
Nivolumab+chemo 1.9 (1.4, 2.7)
Nivolumab+Ipilimumab 1.0 (0.73, 1.4)
Camrelizumab+chemo 0.85 (0.62, 1.2)
Pembrolizumab+chemo 1.3 (0.87, 1.9)
Toripalimab+chemo 1.2 (0.79, 1.7)
0.6
1
3
H
Compared with Chemotherapy
OR (95% CI)
Nivolumab 0.13 (0.078, 0.19)
Camrelizumab 0.36 (0.24, 0.56)
Pembrolizumab 0.35 (0.24, 0.51)
Tislelizumab 0.19 (0.12, 0.28)
Sintilimab 0.44 (0.23, 0.85)
0.07
1
