# Supplementary material for: Comparative efficacy and safety of immunotherapy for patients with advanced or metastatic esophageal squamous cell carcinoma: a systematic review and network Meta-analysis
Source: BMC Cancer. 2022 Sep 17;22:992. doi: 10.1186/s12885-022-10086-5 (PMC9482734; doi:10.1186/s12885-022-10086-5)
Supplement: Supplementary file 8 — Additional file 8. [file 12885_2022_10086_MOESM8_ESM.pdf]

	Decreased appetite	Fatigue	Nausea	Vomiting	Alopecia	Neutropenia	Anemia	Leukopenia	Capillary proliferation	Stomatitis	Diarrhoea	Rash	Malaise	Hypo-thyroidism	Hypo-natremia	Lung infection
**Nivolumab**	8	7	2	NA	1	2	2	1	NA	2	10	11	4	NA	NA	NA
**Camrelizumab**	5	NA	2	1	NA	4	11	7	80	NA	6	NA	10	17	2	NA
**Pembrolizumab**	9	12	7	3	1	1	3	NA	NA	NA	5	NA	7	11	NA	NA
**Sintilimab**	1	5	3	2	NA	5	8	8	NA	NA	4	4	NA	13	13	2
**Nivolumab+Ipilimumab**	6	9	8	6	1	1	4	1	NA	4	10	17	4	13	NA	NA
**Nivolumab+chemo**	41	19	57	17	10	20	29	13	NA	31	19	7	16	NA	NA	NA
**Camrelizumab+chemo**	43	47	50	39	45	67	77	68	80	NA	NA	5	NA	11	NA	5
**Pembrolizumab+chemo**	39	36	62	29	14	36	38	24	NA	26	26	8	12	10	9	5
**Toripalimab+chemo**	39	43	43	40	35	67	78	68	NA	NA	23	22	NA	6	15	10
**Chemotherapy**	63	46	76	41	49	66	79	67	3	16	34	8	24	5	8	3
