# Supplementary material for: Comparative efficacy and safety of immunotherapy for patients with advanced or metastatic esophageal squamous cell carcinoma: a systematic review and network Meta-analysis
Source: BMC Cancer. 2022 Sep 17;22:992. doi: 10.1186/s12885-022-10086-5 (PMC9482734; doi:10.1186/s12885-022-10086-5)
Supplement: Supplementary file 9 — Additional file 9. [file 12885_2022_10086_MOESM9_ESM.pdf]

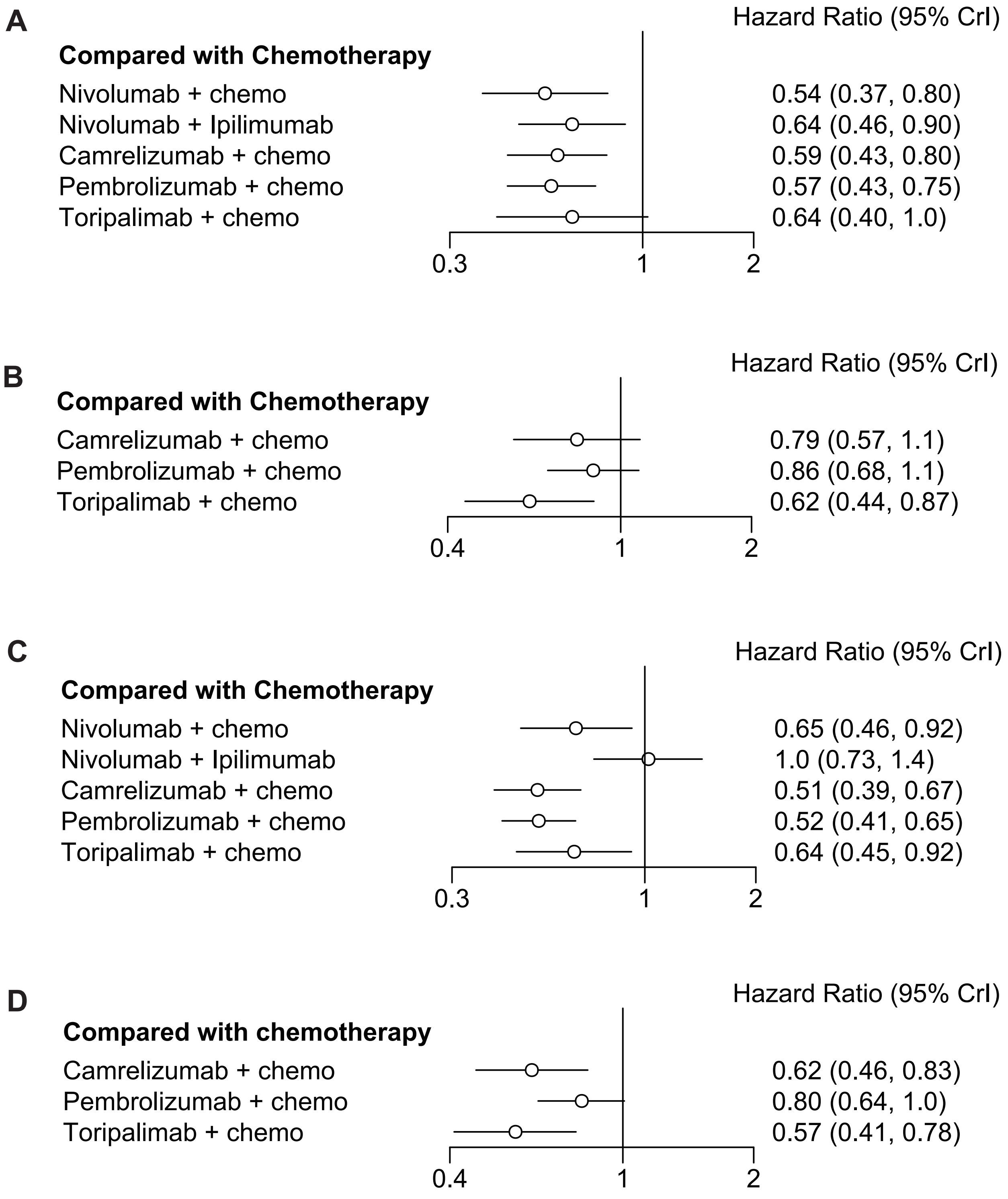

A
Hazard Ratio (95% CrI)
Compared with Chemotherapy
Nivolumab + chemo
Nivolumab + Ipilimumab
Camrelizumab + chemo
Pembrolizumab + chemo
Toripalimab + chemo
0.54 (0.37, 0.80)
0.64 (0.46, 0.90)
0.59 (0.43, 0.80)
0.57 (0.43, 0.75)
0.64 (0.40, 1.0)
0.3
1
2
B
Hazard Ratio (95% CrI)
Compared with Chemotherapy
Camrelizumab + chemo
Pembrolizumab + chemo
Toripalimab + chemo
0.79 (0.57, 1.1)
0.86 (0.68, 1.1)
0.62 (0.44, 0.87)
0.4
1
2
C
Hazard Ratio (95% CrI)
Compared with Chemotherapy
Nivolumab + chemo
Nivolumab + Ipilimumab
Camrelizumab + chemo
Pembrolizumab + chemo
Toripalimab + chemo
0.65 (0.46, 0.92)
1.0 (0.73, 1.4)
0.51 (0.39, 0.67)
0.52 (0.41, 0.65)
0.64 (0.45, 0.92)
0.3
1
2
D
Hazard Ratio (95% CrI)
Compared with chemotherapy
Camrelizumab + chemo
Pembrolizumab + chemo
Toripalimab + chemo
0.62 (0.46, 0.83)
0.80 (0.64, 1.0)
0.57 (0.41, 0.78)
0.4
1
2
