# Supplementary figures and images for: Comparative efficacy and safety of immunotherapy for patients with advanced or metastatic esophageal squamous cell carcinoma: a systematic review and network Meta-analysis
Source: BMC Cancer. 2022 Sep 17;22:992. doi: 10.1186/s12885-022-10086-5 (PMC9482734; doi:10.1186/s12885-022-10086-5)

**A**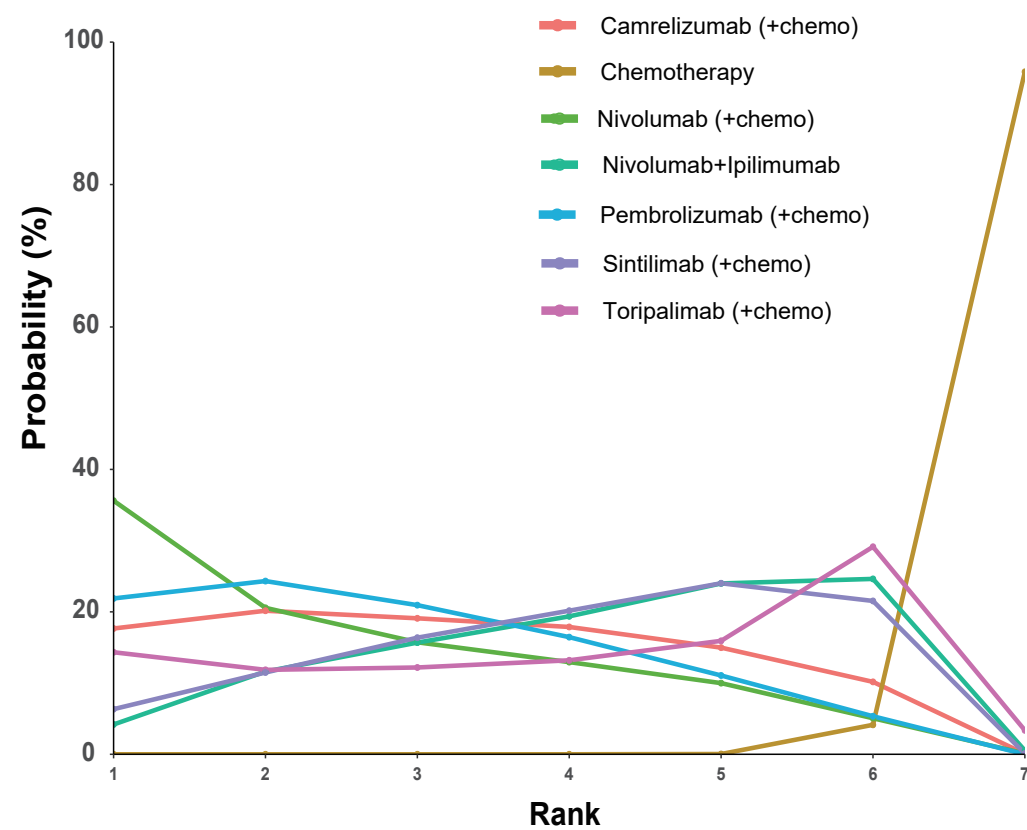**C**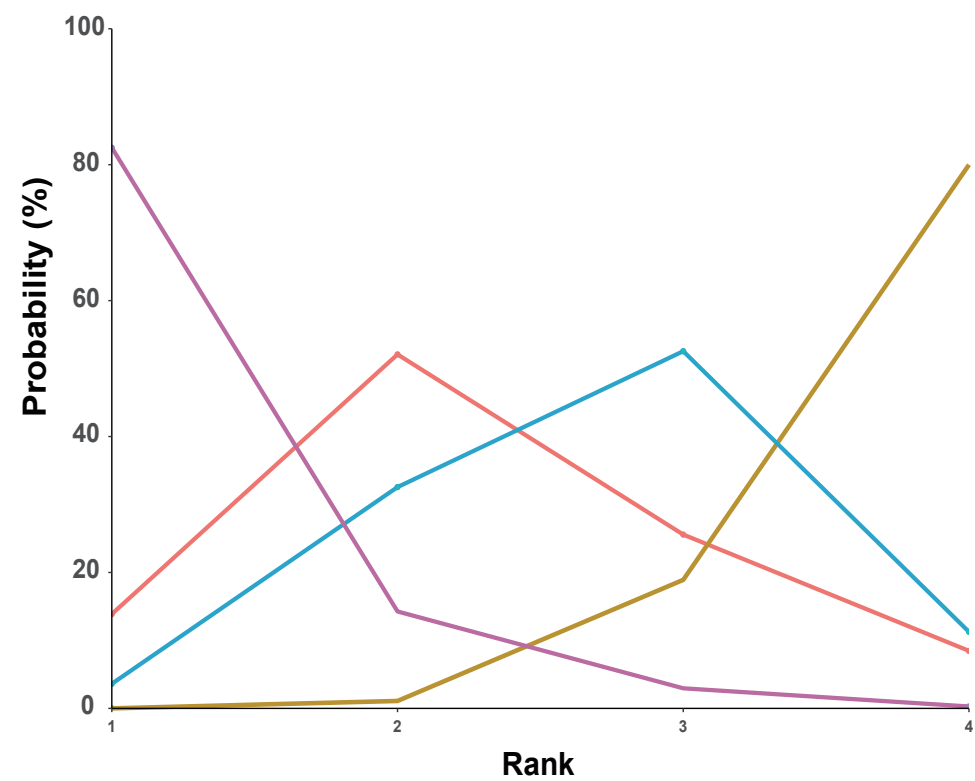**B**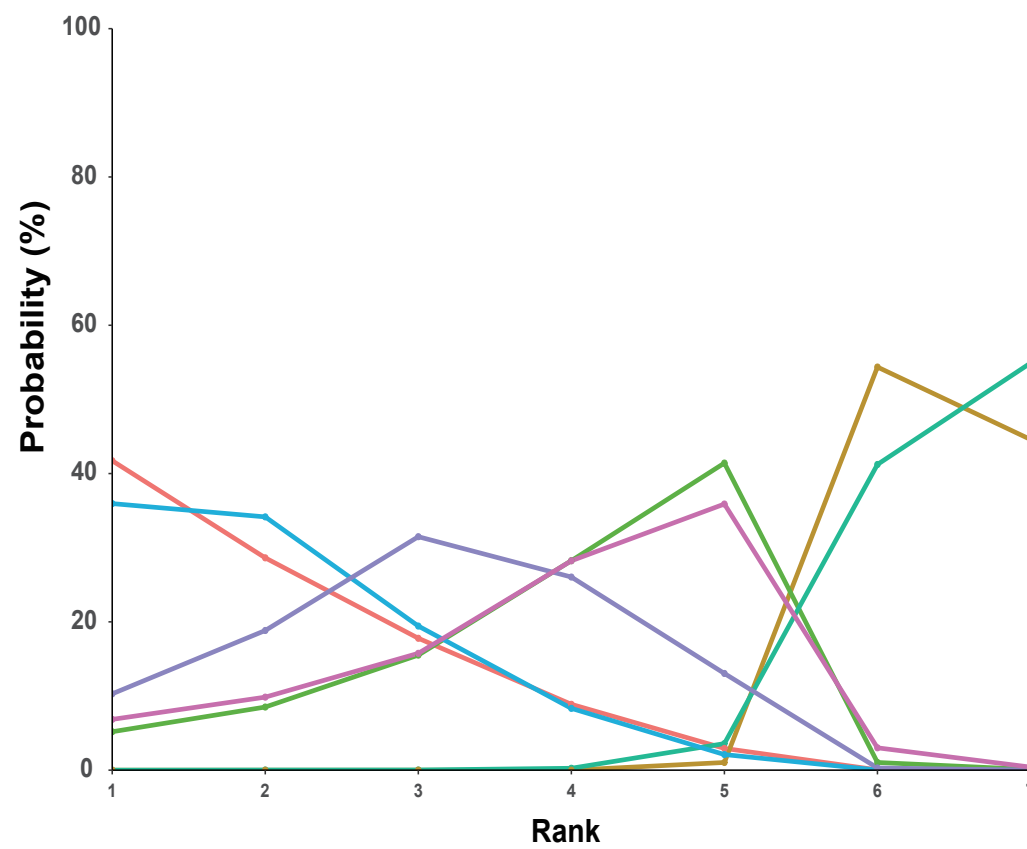**D**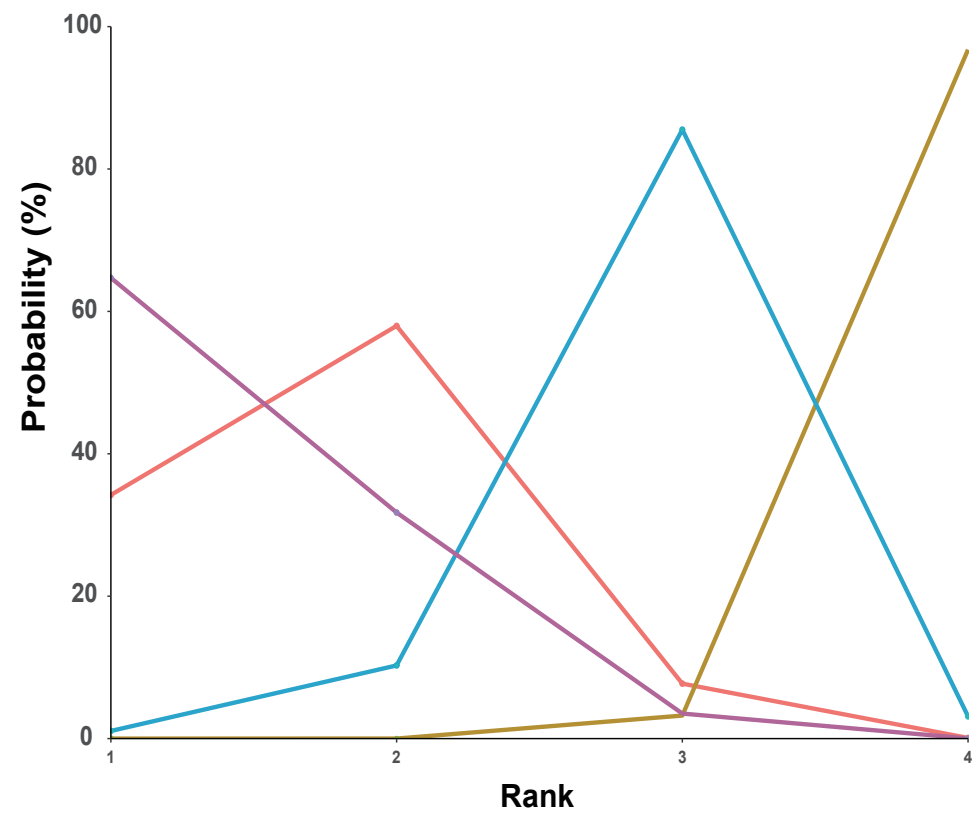

Supplement: Supplementary file 10 — Additional file 10. [file 12885_2022_10086_MOESM10_ESM.pdf]

**A**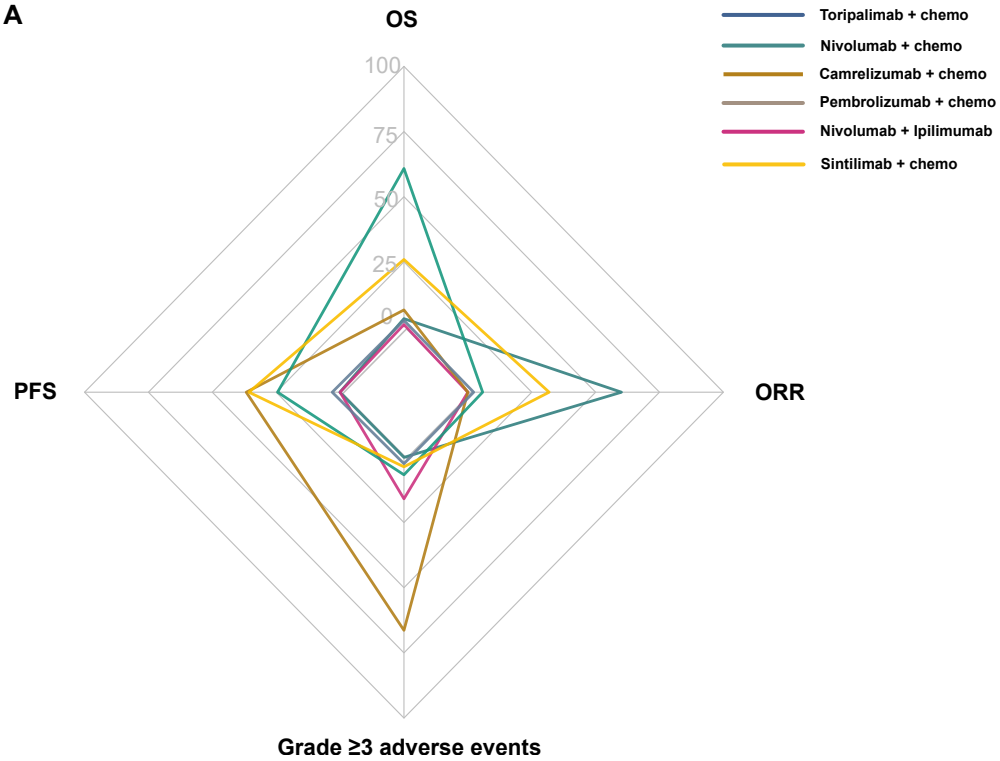**B**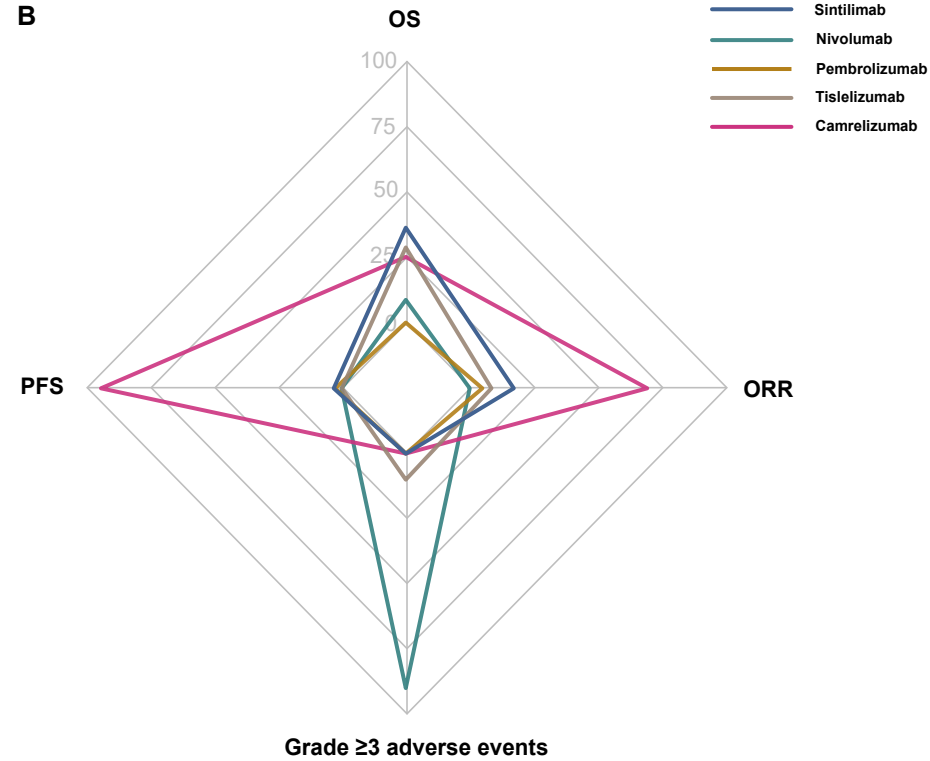

Supplement: Supplementary file 11 — Additional file 11. [file 12885_2022_10086_MOESM11_ESM.pdf]
